# Proteomic profiling of end-stage COVID-19 lung biopsies

**DOI:** 10.1186/s12014-022-09386-6

**Published:** 2022-12-17

**Authors:** Juergen Gindlhuber, Tamara Tomin, Florian Wiesenhofer, Martin Zacharias, Laura Liesinger, Vadim Demichev, Klaus Kratochwill, Gregor Gorkiewicz, Matthias Schittmayer, Ruth Birner-Gruenberger

**Affiliations:** 1 Diagnostic and Research Institute of Pathology, Medical University of Graz, Graz, Austria; 2 Institute of Chemical Technologies and Analytics, Faculty of Technical Chemistry, Technische Universität Wien, Vienna, Austria; 3 Christian Doppler Laboratory for Molecular Stress Research in Peritoneal Dialysis, Department of Pediatrics and Adolescent Medicine, Medical University of Vienna, Vienna, Austria; 4 Division of Pediatric Nephrology and Gastroenterology, Department of Pediatrics and Adolescent Medicine, Comprehensive Center for Pediatrics, Medical University of Vienna, Vienna, Austria; 5 Institute of Biochemistry, Charité-Universitätsmedizin Berlin, Berlin, Germany

**Keywords:** COVID-19, SARS-CoV-2, Lung, Proteomics, Extracellular matrix, Signaling

## Abstract

The outbreak of a novel coronavirus (SARS-CoV-2) in 2019 led to a worldwide pandemic, which remains an integral part of our lives to this day. Coronavirus disease (COVID-19) is a flu like condition, often accompanied by high fever and respiratory distress. In some cases, conjointly with other co-morbidities, COVID-19 can become severe, leading to lung arrest and even death. Although well-known from a clinical standpoint, the mechanistic understanding of lethal COVID-19 is still rudimentary. Studying the pathology and changes on a molecular level associated with the resulting COVID-19 disease is impeded by the highly infectious nature of the virus and the concomitant sampling challenges. We were able to procure COVID-19 post-mortem lung tissue specimens by our collaboration with the BSL-3 laboratory of the Biobanking and BioMolecular resources Research Infrastructure Austria which we subjected to state-of-the-art quantitative proteomic analysis to better understand the pulmonary manifestations of lethal COVID-19. Lung tissue samples from age-matched non-COVID-19 patients who died within the same period were used as controls. Samples were subjected to parallel accumulation–serial fragmentation combined with data-independent acquisition (diaPASEF) on a timsTOF Pro and obtained raw data was processed using DIA-NN software. Here we report that terminal COVID-19 patients display an increase in inflammation, acute immune response and blood clot formation (with concomitant triggering of fibrinolysis). Furthermore, we describe that COVID-19 diseased lungs undergo severe extracellular matrix restructuring, which was corroborated on the histopathological level. However, although undergoing an injury, diseased lungs seem to have impaired proliferative and tissue repair signalling, with several key kinase-mediated signalling pathways being less active. This might provide a mechanistic link to post-acute sequelae of COVID-19 (PASC; “Long COVID”). Overall, we emphasize the importance of histopathological patient stratification when interpreting molecular COVID-19 data.

**Supplementary Information:**

The online version contains supplementary material available at 10.1186/s12014-022-09386-6.

## Introduction

Spreading of the novel coronavirus (severe acute respiratory syndrome coronavirus 2 (SARS-CoV-2)) resulted in a worldwide pandemic and health emergency [1, 2]. SARS-CoV-2 induced coronavirus disease (COVID-19) is most frequently manifested with mild to severe symptoms, including fever, fatigue, cough, sore throat and a number of other clinical presentations [3]. However, in more complicated cases, COVID-19 is associated with acute respiratory distress syndrome (ARDS), which can result in respiratory failure and even death [4, 5]. As COVID-19 is primarily a respiratory condition, lungs represent the most affected organ. Correspondingly, autopsy reports of end-stage COVID-19 patients indicate diffuse alveolar damage (DAD) as the major pathology. Furthermore, a considerable amount of thrombotic material has been observed, causing pulmonary infarction in up to 73% of cases [6]. Despite functioning intensive care unit [7] (ICU) therapeutic approaches such as dexamethasone treatment [8], mortality in patients that require mechanical lung ventilation remains at 20–40% [4, 8].

In order to further dissect the underlying pathomechanism of a fatal SARS-CoV-2 infection on the human lungs, we carried out a comprehensive proteomic profiling of lung tissue (two individual samples per patient) obtained from six consecutive end-stage COVID-19 cases during the first pandemic wave, preventing any potential selection bias. We compared them to six age-matched autopsy cases without SARS-CoV-2 infection and without recorded inflammatory conditions. Tissue pieces were collected at the point of autopsy and directly transferred into lysis buffer for proteomic analysis, reducing any potential sample storage bias. The selection of sampling areas within COVID-19 lungs was guided by a careful macroscopic examination, specifying regions with pronounced virus-induced changes.

Our data confirms that the end-stage COVID-19 lung is characterized by increased expression of proteins involved in complement system activation, clot formation and consequent activation of fibrinolysis that allow for clot clearance, as well as other pro-inflammatory pathways. Deceased COVID-19 patients displayed a prominent reduction of proteins responsible for extracellular remodelling and integrity of the basal membrane, especially laminins. Furthermore, pathway analysis predicted a decreased activation of a number of different signalling pathways in the COVID-19 patients, namely those involved in cellular proliferation and tissue repair mechanisms. Lastly, we could demonstrate that different COVID-19 related death causes (diffuse alveolar damage versus secondary pneumonia) are reflected by distinct proteomics signatures, emphasizing the importance of histopathological patient stratification when interpreting molecular COVID-19 data. Overall, this work provides a comprehensive overview of proteome perturbances caused by COVID-19 in failing lung tissue and as such establishes comprehensive ground for further therapeutic studies.

## Materials and methods

If not stated otherwise, all chemicals were purchased from Sigma-Aldrich.

### Patient information and sample collection

Lung tissue specimens were collected from six consecutive SARS-CoV-2 positive patients during the first pandemic wave and from six age-matched control patients without SARS-CoV-2 infection and without recorded inflammatory conditions (Table 1). Two samples were taken from each patient either from macroscopically affected lung tissue (SARS-CoV-2 cohort) or from normal homogeneous lung parenchyma (control cohort). Additional material for histology was collected from the same areas. An extended list of patients’ metadata is included in the (Additional file 2: Table S1). Of note, the COVID-19 cohort in the present study corresponds to cases 15 to 20 in our initial autopsy study where comprehensive metadata can be found [9].

**Table 1:** Overview of the supporting patient information

Case ID	Group	Age (years)	Sex	Cause of death	First positive SARS-CoV-2 PCR (days)	PM interval (hours)	Antibiotic therapy	Oxygen therapy	ICU
P1	COVID-19	80	Female	Pneumonia	7	46	Yes	Yes	No
P2	COVID-19	81	Female	Pneumonia	11	23	No	Yes	No
P3	COVID-19	80	Male	Pneumonia	6	7	Yes	Yes	No
P4	COVID-19	54	Male	DAD + pneumonia	23	14	Yes	Yes	Yes
P5	COVID-19	65	Male	DAD	34	57	Yes	Yes	Yes
P6	COVID-19	79	Female	DAD	2	12	No	Yes	Yes
HP1	Control	58	Male	Multiorgan failure	Neg	48	Yes	Yes	No
HP2	Control	61	Male	Ruptured aortic aneurysm	Neg	61	No	Yes	No
HP3	Control	82	Male	Myocardial infarction	Neg	39	Yes	Yes	Yes
HP4	Control	82	Female	Myocardial infarction	Neg	102	No	No	No
HP5	Control	74	Male	Metastatic cancer	Neg	28	No	No	No
HP6	Control	82	Male	Pulmonary embolism	Neg	22	Yes	Yes	Yes

First positive SARS-CoV-2 PCR represents the time between PCR test and death. Post-mortem interval (PM) are the hours after death until autopsy/tissue collection. *DAD* diffuse alveolar damage, *ARDS* acute respiratory distress syndrome, *ICU* intensive care unit, *PCR* Polymerase chain reaction.

### Proteomics sample preparation

The tissue pieces measuring approximately 3 × 3 ×  3 mm in volume were collected in BeadBug™ 2.0 ml tubes containing 2.8 mm stainless steel beads and filled with 600 µl of 0.1 M Tris–HCl at a pH of 7.6 buffer containing 2% sodium dodecyl sulphate (SDS) and 10 mM Tris(2-carboxyethyl)phosphine. Collected biopsies were homogenized using a MagNA Lyser (Roche, USA) bead mill set to 6500 rpm for 3 × 25 s. Samples were cooled on ice after each run to avoid excessive heating of the sample. A 30 min 3500 g centrifugation step at 4 °C was performed to remove all insoluble cellular debris. Protein content was estimated using bicinchoninic acid assay (Thermo Fisher Scientific, USA), after which 100 µg of protein per sample was precipitated overnight with three volumes of acetone. Protein pellets were re-dissolved in 25% trifluoroethanol (in 100 mM Tris pH 8.5), diluted to 10% trifluoroethanol with ammonium bicarbonate and digested overnight with trypsin (Thermo Fisher Scientific, USA). Consequently, 4 µg of digest was offline desalted using in-house made stage tips [11] and 300 ng per sample was used for liquid chromatography tandem mass spectrometry (LC–MS/MS) analysis.

### LC–MS/MS analysis

Proteins were separated on the Ultimate 3000 RCS Nano Dionex system equipped with an Ionopticks Aurora Series UHPLC C18 column (250 mm × 75 µm, 1.6 µm) (Ionopticks, Australia), with solvent A being 0.1% formic acid in water and solvent B acetonitrile containing 0.1% formic acid. Total LC–MS/MS run time per sample was 136.5 min with the following gradient: 0–5.5 min: 2% B; 5.5–65.5 min: 2–17% B; 65.5–95.5 min: 25–37% B, 105.5–115.5 min: 37–95% B, 115.5–125.5 min: 95% B; 125.5–126.5 min: 95–2% B; 126.5–136.5 min: 2% B at a flow rate of 400 nl/min and 40 °C. The timsTOF Pro mass spectrometer (Bruker Daltonics, Germany) was operated in positive mode with enabled trapped ion mobility spectrometry (TIMS) at 100% duty cycle (100 ms ramp time). Source capillary voltage was set to 1500 V and dry gas flow to 3 L/min at 180 °C. Scan mode was set to data independent parallel accumulation–serial fragmentation (diaPASEF) using parameters previously described [12]. In brief, 32 isolation windows of 26 m/z width spanning from from m/z 400 to 1,200 were defined, with m/z of 1 overlap between the windows (on each side of a given window). After an MS1 scan, 2 isolation windows were fragmented per TIMS ramp from both sides of the mass range (e.g. m/z 400–426 and 800–826). The collision energy was set to rise linearly over the covered mobility range (for 1/K0 values between 0.6 and 1.6, 20 to 59 eV correspondingly). Total resulting DIA cycle time was estimated to be 1.7 s.

### Data processing

Raw data files were analysed and proteins were quantified using DIA-NN software (version 1.7.13 beta 12 [13, 14]). The SwissProt human proteome database in fasta format (containing common contaminants; downloaded on 16.04.2019, 20,467 sequences) was used for a library-free search with FDR set to 1%. Deep learning-based spectra and retention time prediction was enabled, minimum fragment m/z was set to 200 and max fragment m/z set to 1800. N-terminal methionine excision was enabled and maximum number of trypsin missed cleavages set to 1. Minimum peptide length was set to 7 and the maximum to 30 amino acids. Cysteine carbamidomethylation was set as a fixed and methionine oxidation as a variable modification. Mass accuracy was fixed to 10 ppm for both MS1 and MS2. The mass spectrometry proteomics datasets (including the DIA-NN version used to process the data) have been deposited to the ProteomeXchange Consortium via the PRIDE partner repository [15] with the dataset identifier PXD030009.

### Statistical analysis and data visualization

For data analysis, tissue pieces collected from the same patient were considered as technical duplicates and the mean value per each protein was taken whenever possible. Distribution as well as contribution of individual replicates to the pool of quantified proteins is displayed in the (Additional file 1: Fig. S1A). For most of the samples, the majority of proteins were shared between the two replicates.

The dataset was filtered to keep only those proteins with a minimum of four valid values in each of the two groups and the statistical analysis was performed using the limma R-package [16].

For the analysis of the cause of COVID-19 related death on the lung proteome, Perseus 1.6.14.0 was used [17]. Briefly, COVID-19 samples were classified into two groups based on histopathology (DAD versus pneumonia). The data matrix was then filtered to keep only those proteins measured in at least four replicates of both subgroups. The resulting list of proteins was then subjected to Student’s t-testing. Due to small number of samples, statistics was carried out both with and without multi-testing correction. To gain an insight into potentially affected biological process, all significantly changed proteins before multi-testing correction (Student’s t-test p-value < 0.05) were used for the enrichment analysis as described below.

### Reactome pathway, gene ontology and network analysis

Reactome pathway analysis was carried out using Reactome Pathway Browser (v3.7). Protein network analysis as well as gene ontology enrichment of biological processes (GOBP) was performed using the String database (v11.0) plug-in in Cytoscape (v3.8.2). Visualization of the enrichment analysis was done in Enrichment Map (v 3.3.1). For String network analysis (Fig. 3A) a high confidence cut-off for protein interactions was set (0.7; range 0–1). In both cases (Reactome and String analysis), significantly changed proteins (limma dynamic p-value < 0.05,) were used as input. In addition, for a more comprehensive overview of metabolic and pathological alterations, all significantly changed proteins before multi-testing correction (Student’s t-test p-value < 0.05) were subjected to Ingenuity Pathway Analysis (IPA, QIAGEN, Germany). IPA provides enrichment tests for canonical pathways, diseases and functions and possible upstream regulatory elements of differentially expressed proteins.

In case of DAD versus pneumonia, proteins with a Student’s t-test p-value < 0.05 and a log_2_ fold change of ± 0.5 (between the two conditions) were used for GOBP enrichment analysis in String (v11.5). The false discovery rate for all enrichment analyses was less than 5%.

### Movat pentachrome stain

2–4 µm slices of formalin fixed paraffin embedded tissue were subjected to a Movat pentachrome stain to visualize and differentiate extra cellular matrix components. First slides were brought to water, stained with Verhoeff's elastic solution (hematoxylin & ferric chloride solution Gatt-Koller, Austria; Iodine & Potassiumiodate Sigma-Aldrich, Austria) for 1.5 h and rinsed in warm running water for 20 min. After a quick rinse with distilled water, slides were dipped two times into 2% ferric chloride for differentiation. The process was closely monitored and either stopped with distilled water or if necessary repeated to obtain a prominent staining. After two dips in 5% sodium thiosulfate and 3% acetic acid (Gatt-Koller, Austria), separated by rinsing in cold water for 5 min, staining with alcian blue was performed for 30 min. Samples were again rinsed with warm running water for 10 min, one more time with distilled water and then stained with croceine scarlet–acid fuchsin (Chroma-Waldeck GmbH, Germany) for 2 min. After rinsing in distilled water for three times (exchanging the water each time), another rinse with 1% acetic acid water was performed, prior to differentiation with 2 dips in 5% aqueous phosphotungstic acid. The differentiation was stopped with a quick rinse in 1% glacial acetic acid to prevent loss of fibrin stain. For the final step, slides were brought into absolute alcohol, exchanged three times, and stained with Safron du Gatinais (alcoholic safran solution; Chroma-Waldeck GmbH, Germany). Slides were rinsed again in absolute alcohol, exchanged three times, followed by clearing and mounting.

## Results

### COVID-19 lungs show a prominent upregulation of fibrinolysis and immune/stress response

The COVID-19 cohort consisted of 3 males and 3 females with an age range from 54 to 81 (median 79.5 years). Time from first positive SARS-CoV-2 (ante mortem) PCR to death ranged from 2 to 34 days (median: 9 days) and the post-mortem interval ranged from 7 to 57 h (median 18.5 h). Details about patient metadata are provided in Table 1 and (Additional file 2: Table S1). For both COVID-19 patients and controls, two individual lung tissue pieces were collected at the point of autopsy. Tissue pieces originating from the same patient (Additional file 1: Fig. S1A) were treated as replicates, were processed as described in the materials and methods section and then subjected to comprehensive proteomics analysis. The resulting protein list containing the mean quantitation data from two technical replicates (or the single quantitative value if observed in only one technical replicate) was then filtered to maintain only those proteins with reported values in at least four samples of each group (COVID-19 and control).

**Fig. 1:**
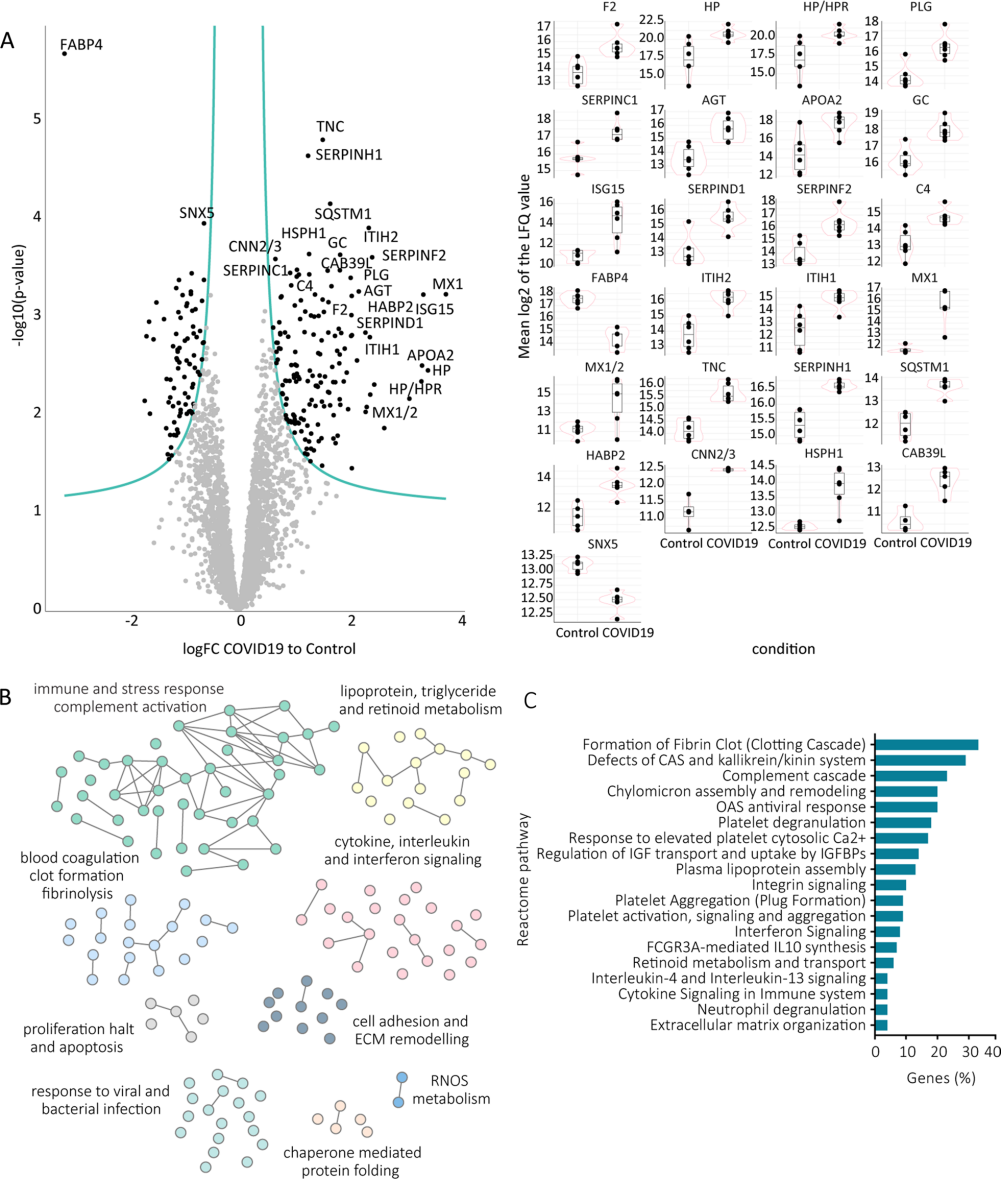
The most upregulated proteins in lung tissues of terminal COVID-19 patients are involved in immune and stress response, extracellular matrix remodelling, as well as clot formation and fibrinolysis. **A** Volcano plot of the LFQ diaPASEF dataset after limma analysis (limma dynamic p-value < 0.05) including box dot plots of the most prominently changed proteins. **B** Grouping of selected significantly enriched biological processes (limma dynamic p-value < 0.05) based on similarity after gene ontology enrichment analysis using String and Enrichment map in Cytoscape with significantly more abundant proteins in COVID-19 (limma dynamic p-value < 0.05, fold change to control > 1.5) as input. **C** Selected significantly enriched Reactome pathways (enrichment FDR corrected p-value < 0.05) with the same input protein list (as for **B**)

This produced a matrix of 3431 proteins which was then subjected to statistical analysis using a linear model approach [16]. All 3431 proteins including the fold changes (COVID-19 *versus* control) and statistics are listed in (Additional file 3: Table S2). At all times, a minimum alpha level of 0.05 and a minimum fold-change of 1.5 was maintained and borderline significant proteins with low fold-change were excluded from the analysis, as indicated by the dynamic cut-off (Fig. 1A). This approach resulted in identification of 239 significant differentially abundant proteins between COVID-19 and control lung samples (Fig. 1A). The significantly more abundant proteins in COVID-19 samples (dynamic p-value cut off < 0.05 and fold change to control > 1.5) were then used for enrichment analysis of either biological processes (using String database and Cytoscape for visualization; Fig. 1B) or metabolic and signalling pathways (using Reactome pathway analysis; Fig. 1C).

Among the most prominently upregulated proteins in COVID-19 lungs were members of the complement activation cascade (e.g. C4, C3, C5, B, C2, H, C9), different interferon-induced proteins (e.g. Interferon-induced GTP-binding protein Mx1 (MX1)) as well as inter-alpha-trypsin inhibitor family (ITIH) proteins, known acute phase proteins that are upregulated during inflammation [18]. Also other inflammatory proteins were more abundant in COVID-19 lungs, including different isoforms of alpha-1-acid glycoprotein [19] as well as vitamin D binding protein (GC), which not only transports vitamin D but also acts as immune system activator [20, 21]. In addition, we report a prominent upregulation of proteins active in blood clot formation, including prothrombin (F2), coagulation factor XII (F12), antithrombin-III (SERPINC1), plasminogen (PLG) and others (Additional file 3: Table S2).

Correspondingly, both String GO and Reactome pathway analyses resulted in the enrichment of the same major processes/pathways, including fibrin clot formation, platelet activation, immune activation as well as cytokine, interleukin and interferon signalling (Fig. 1B, C). In addition, several regulators of the extracellular matrix (ECM) organization and structure seemed to be affected by SARS-CoV-2 infection (Fig. 1B, C). Extracellular proteins which are higher in the COVID-19 group were mainly involved in processes of clot formation (fibrinogens and plasminogens), lipid transport (apolipoproteins) as well as *de-novo* collagen synthesis (Serpin H1 and prolyl 4-hydroxylase subunit alpha-1; Additional files 3, 4). However, while these extracellular proteins were higher expressed in COVID-19 patients, expression of other ECM proteins appeared to be completely abolished in the terminal infected lungs, as will be discussed in the following subchapter. The list of all enriched biological processes and pathways (with proteins significantly more abundant in COVID-19 samples as input (limma dynamic p-value < 0.05)) can be found in (Additional file 4: Table S3).

### Expression of prominent ECM constituents and regulators is diminished in COVID-19 affected lungs

**Fig. 2:**
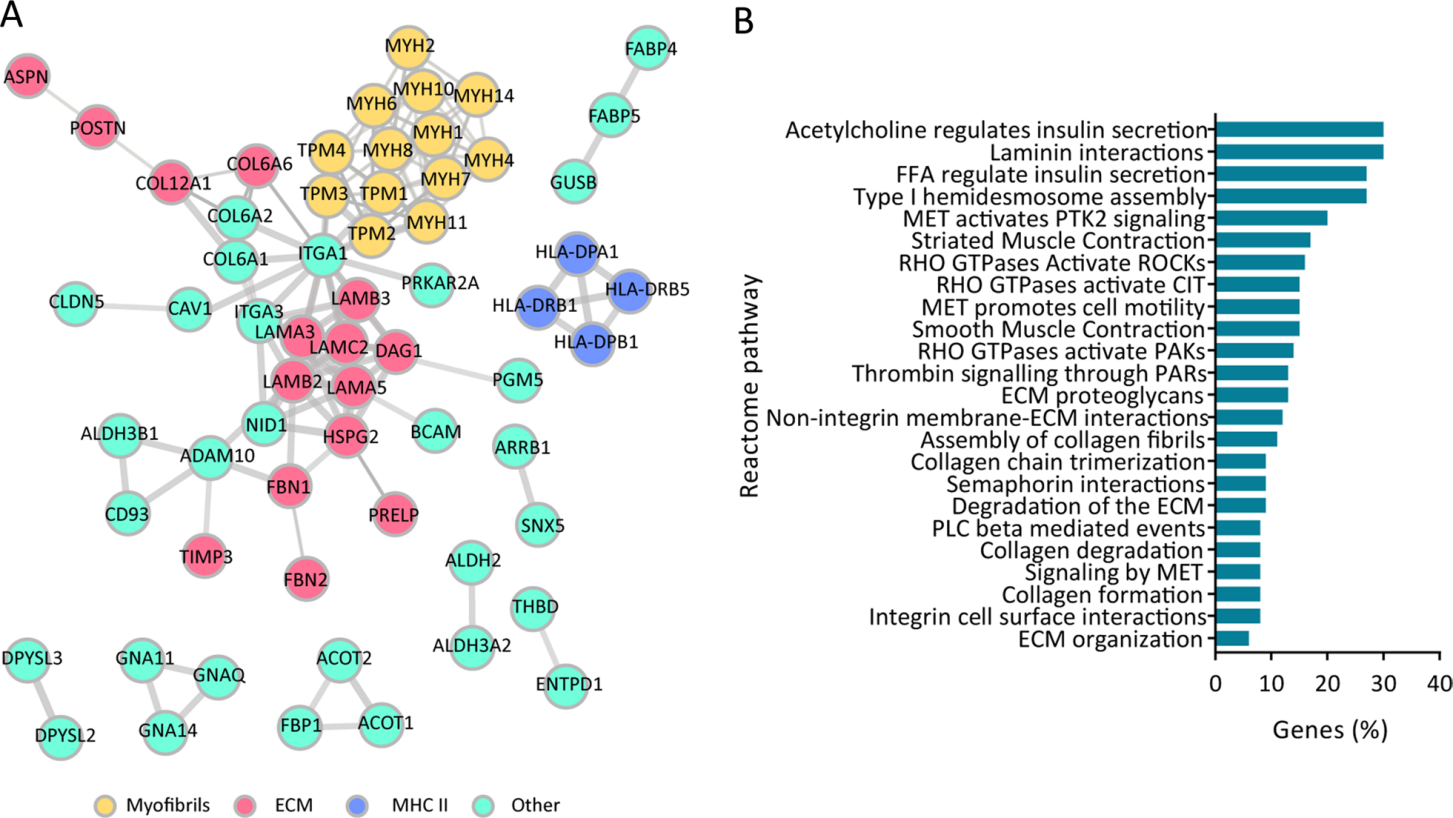
The most prominently downregulated proteins in COVID-19 patient lung tissue mainly belong to the ECM constituent and fibril/filament formation. **A** String protein interaction network of significantly downregulated proteins in COVID-19 patients (dynamic p-value < 0.05; interaction confidence level 0.7 (high)); **B** Data from String was further corroborated with IPA predicted “activated” or “deactivated” significantly enriched diseases and functions in COVID-19 patients with significantly altered proteins (p-value < 0.05) as input. Higher Z-score represents predicted increase of the given pathway/function in COVID-19 patients while lower Z-score predicts a decrease (Z-Score cut-off ± 1.5)

Interestingly, it seems that while SARS-CoV-2 infected lungs cope with a cytokine storm, complement system activation and increased clot formation (Figs. 1B, C and 2B), they also undergo severe ECM restructuring. Correspondingly, quantitative proteomic profiles of the infected lungs revealed reduced abundance of a number of different ECM proteins, including constituents of the basement membrane as well as different types of collagen and fibrillin (Fig. 2A, Additional file 3: Table S2). In line with this, Ingenuity Pathway Analysis (IPA) of significantly altered proteins resulted in the enrichment of cell migration and cell movement processes, which are known to be associated with structural changes of the ECM (Fig. 2B).

**Fig. 3:**
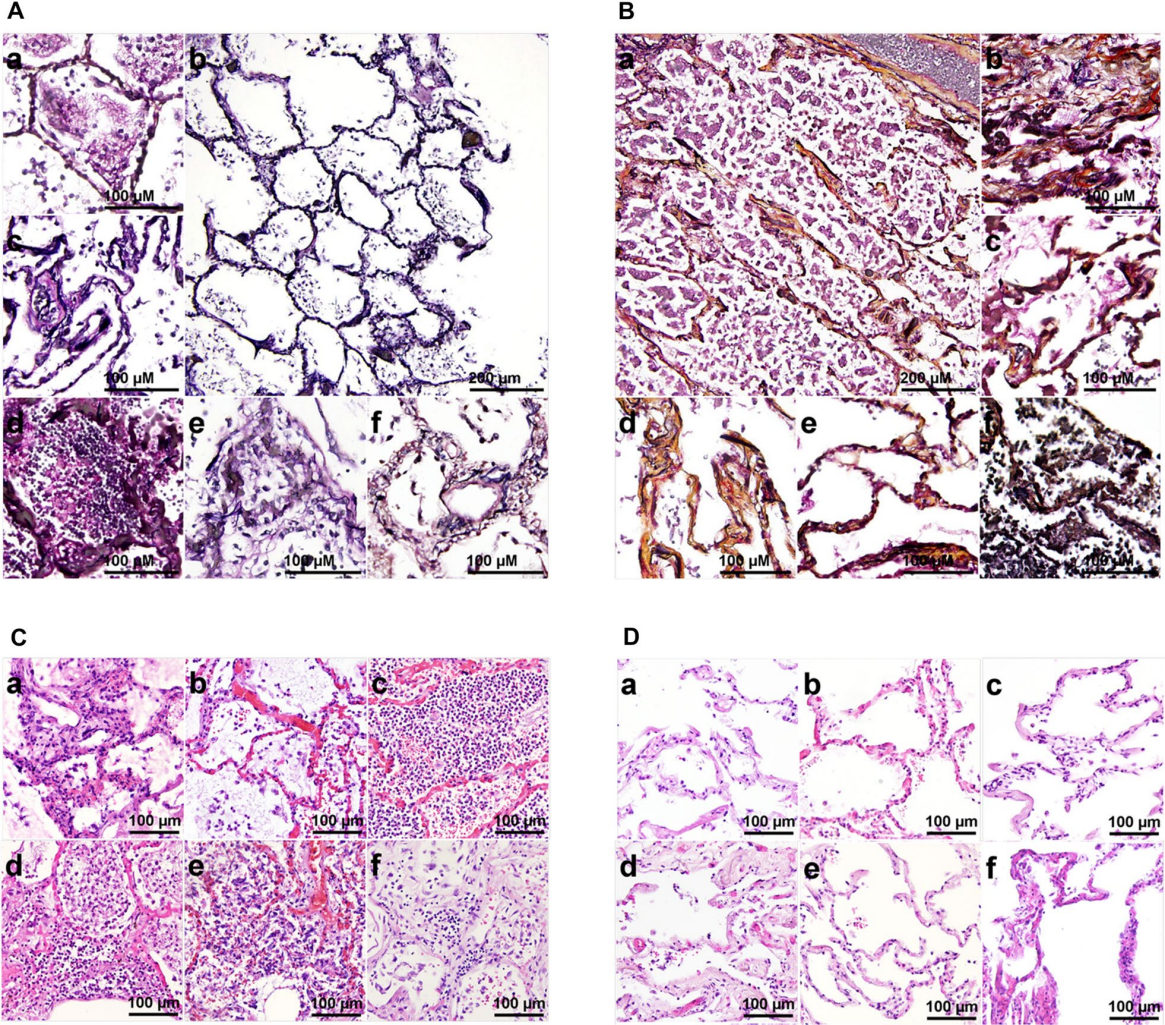
Histopathology of COVID-19 patient lung tissue reveals loss of extracellular matrix and prominent infiltration of immune cells**.** **A** Movat pentachrome stain of lung tissue slices from COVID-19 patients. **a**–**e** 20 × images, **b** 10 × overview. Whilst overall tissue structure is still well defined, none stained positive for collagen and reticular fibres. **B** **a** 10 × overview, **b**–**f** 20 × images of healthy lung tissue controls. Except **f** all of the healthy controls display a strong positive staining result (brownish yellow) for collagen and reticular fibres. The reduced signal in f could be explained by an incidental focal inflammation. **C** **a**–**f** 20 × images of H&E stains of COVID-19 samples. Samples display a varying amount of infiltrated immune cells, from minor, early onset inflammation (**a**, **b**, **f**) to advanced inflammation (**d**, **e**) to alveoli filled with infiltrate (**c**). **D** **a**–**f** 20 × images H&E stains of healthy control patients display clinically unremarkable lung parenchyma. Displayed images are representatives of 10 pictures taken per each of the six COVID-19 and control patient analysed in this study

Proteomics findings were further corroborated by histopathological analysis after staining using the pentachrome method, which allows for simultaneous staining of both collagen (yellow) and sulfated mucopolysaccharides (light blue), in addition to nuclei (black), muscle (red) and elastin (purple) [22]. As visible on Fig. 3A, COVID-19 affected lungs have almost no residual yellow colouring, suggesting massive loss of collagen structure. In addition, structural organisation of elastic fibres also seems deregulated in diseased lungs.

In addition to reduced collagen and fibril expression, our COVID-19 lungs also display decreased abundance of a number of different myosin and tropomyosin isoforms, as well as laminins, which are critical components of the basement membrane. Viral penetration in lungs is known to cause cytoskeleton rearrangement [23] and consequent infection can lead to the disruption of alveolar-capillary barrier, leading to lung injury and reduced gas flow [24, 25]. One of the key protein groups responsible for maintaining the integrity of the barrier (through formation of tight junctions) are cadherins, especially E-cadherin (Cadherin-1) [24]. Correspondingly, in our dataset we observe a trend towards lower expression of a number of cadherin isoforms, including cadherin-1, 5, 13 as well protocadherin-1 (Additional file 1: Fig. S1 and Additional file 3: Table S2, respectively), suggesting a tight-junction breach in the lungs infected by SARS-CoV-2. As a result of such injury, structural organization of the underlying laminin-rich basement membrane can occur and recent reports indeed describe the loss of laminin expression and disruption of the laminin structural arrangements in lungs of COVID-19 patients [26]. Our study further corroborates these findings, as next to reduced abundance of cadherins, laminins seem to be diminished in lungs of COVID-19 patients (Fig. 2; Additional file 3: Table S2).

Lastly, lungs of terminally ill COVID-19 patients also display a prominent reduction in the expression of several members of the major histocompatibility complex (MHC) class II, including HLA-DPA1, HLA-DRB1 and HLA-DRB5, all of which are reported to be reduced in antigen presenting cells of critically ill COVID-19 patients [27].

### Signalling is prominently affected as a consequence of SARS-CoV-2 infection

**Fig. 4:**
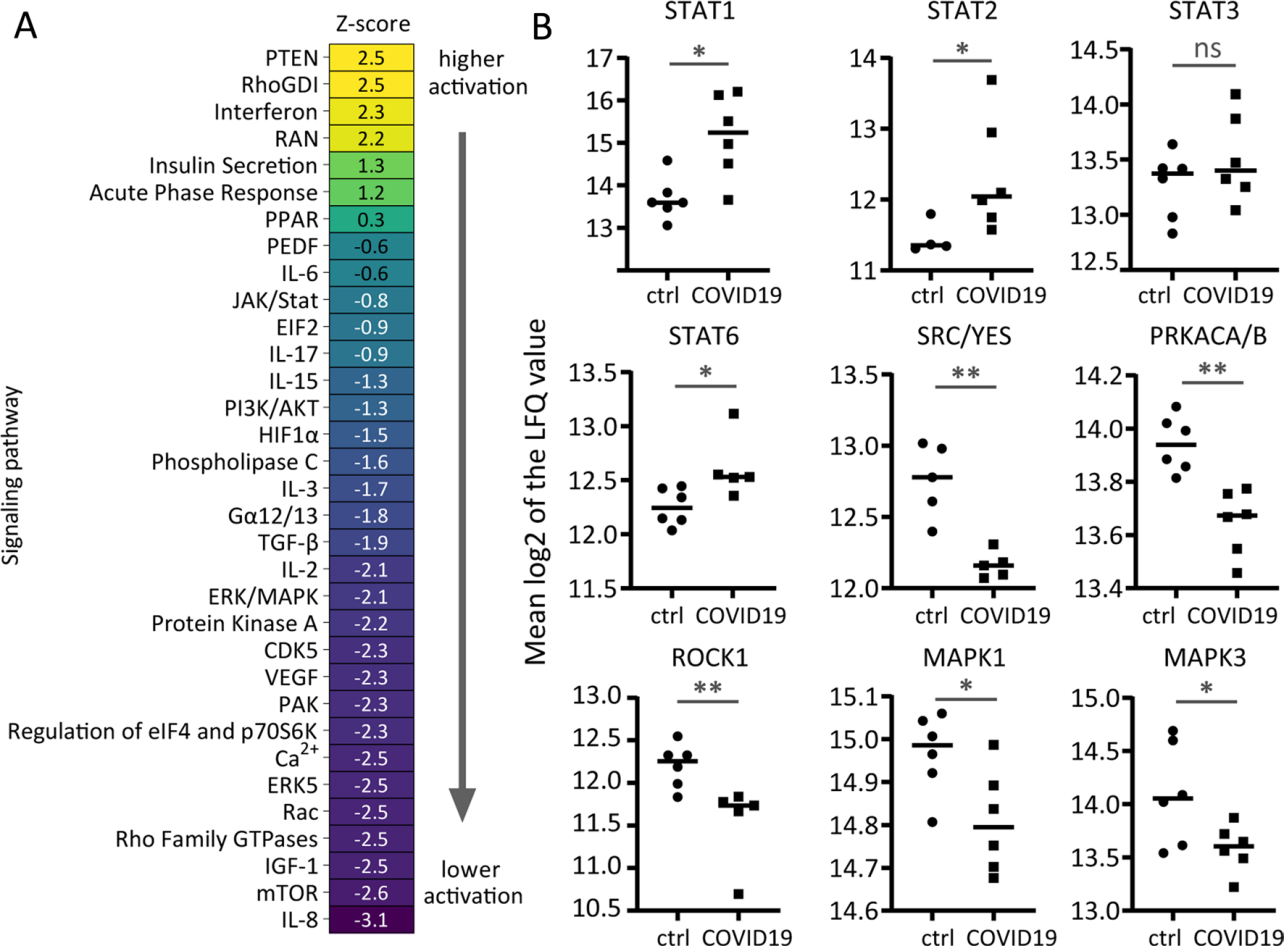
Lungs of COVID-19 patients depict pronounced changes in global, kinase mediated signalling. **A** IPA analysis of canonical pathways with significantly altered proteins before multi-testing correction (p-value < 0.05) as input revealed prediction of lower activation of a number of different, major kinase signalling pathways. Higher Z-score (marked in yellow) predicted activation, while lower Z-score (purple colour) represents prediction of a lower activation of a given pathway in lungs of COVID-19 patients. FDR control on pathway level (enrichment FDR corrected p-value < 0.05). **B** Lungs of COVID-19 patients show reduced abundance of several key kinases but prominent activation of STATs, typical mediators of inflammation (*Student’s t-test p-value < 0.05, ** Student’s t-test p-value < 0.01)

As expected upon viral infection and according to the IPA analysis of canonical pathways, COVID-19 affected lungs show a strong activation of interferon and acute phase response signalling (Fig. 4A), accompanied by the higher expression of a number of interferon-induced proteins (Additional files 3 and 5) as well as signal transducers and activators of transcription (STATs; Additional files 3 and 5, Fig. 4B), known acute inflammatory responders [28].

In addition, severe SARS-CoV-2 infection seems to induce host’s tumor suppressor phosphatase and tensin homolog (PTEN) signalling (Fig. 4A). PTEN is a known antagonist of PI3K/AKT axis and an upstream negative regulator of mammalian target of rapamycin (mTOR) signalling [29], both of which were predicted to be downregulated in our dataset (Fig. 4A). Such an overexpression of PTEN followed by concomitant inhibition of key cellular responsive pathways can be detrimental for the antiviral response, as it leads to suppression of antibody production and consequently a worse outcome [30].

However, not only downstream targets of PTEN are affected by SARS-CoV-2 infection. A number of signalling pathways was predicted as deactivated in COVID-19 lungs (Fig. 4A), including other major kinases responsible for cellular proliferation and cell cycle progression (Fig. 4A, B).

These findings are in line with a recent large time-course phosphoproteomics study of SARS-CoV-2 infection in Vero E6 cells (cells highly susceptible to SARS-CoV-2 infection), which described that the viral infection promotes the host’s p38-MAPK cascade while shutting down key mitotic kinases, including phosphoinositide-3-kinase (PI3K), RAC-alpha serine/threonine-protein kinase 1 and 2 (AKT/2), cAMP-dependent protein kinase (PRKACA/B), Rho-associated protein kinase (ROCK1/2) and others [31]. It seems that together with the overexpression of PTEN, SARS-CoV-2 infection indeed leads to a full proliferation halt in the hosts’ lungs.

Furthermore, interleukin signalling is also predicted to be decreased in lungs of terminal COVID-19 cases (Fig. 4A). Although IL-6 has been reported to be increased in plasma of COVID-19 patients [32, 33] and higher neutrophilic IL-8 expression was observed in severe COVID-19 cases [34], we did not detect such trends with our proteomics approach locally in the lung tissue post mortem. However, pre-mortem blood draw of the six COVID cases in our study showed elevated IL-6 serum levels [9]. Overall observed deactivation of various interleukin pathways in COVID-19 patients might be partially due to anti-IL-6 treatment (one patient) and/or potential glucocorticoid treatment (at least two COVID-19 patients received prednisolone while at the ICU). Glucocorticoids (including dexamethasone) are known to supress interleukin signalling [35–37].

Lastly, insulin signalling is predicted to be activated in the lungs of COVID-19 patients (Fig. 4A). This is not surprising and can almost act as a positive control of this study, as four out of six COVID-19 patients (70%) were diabetic, but none of the controls (Additional file 2: Table S1). Correspondingly, glucose metabolic disorder was the most probable predicted disease in the IPA analysis (see Fig. 2B).

### Histopathological stratification of COVID-19 cases is reflected by distinct proteomics signatures

**Fig. 5:**
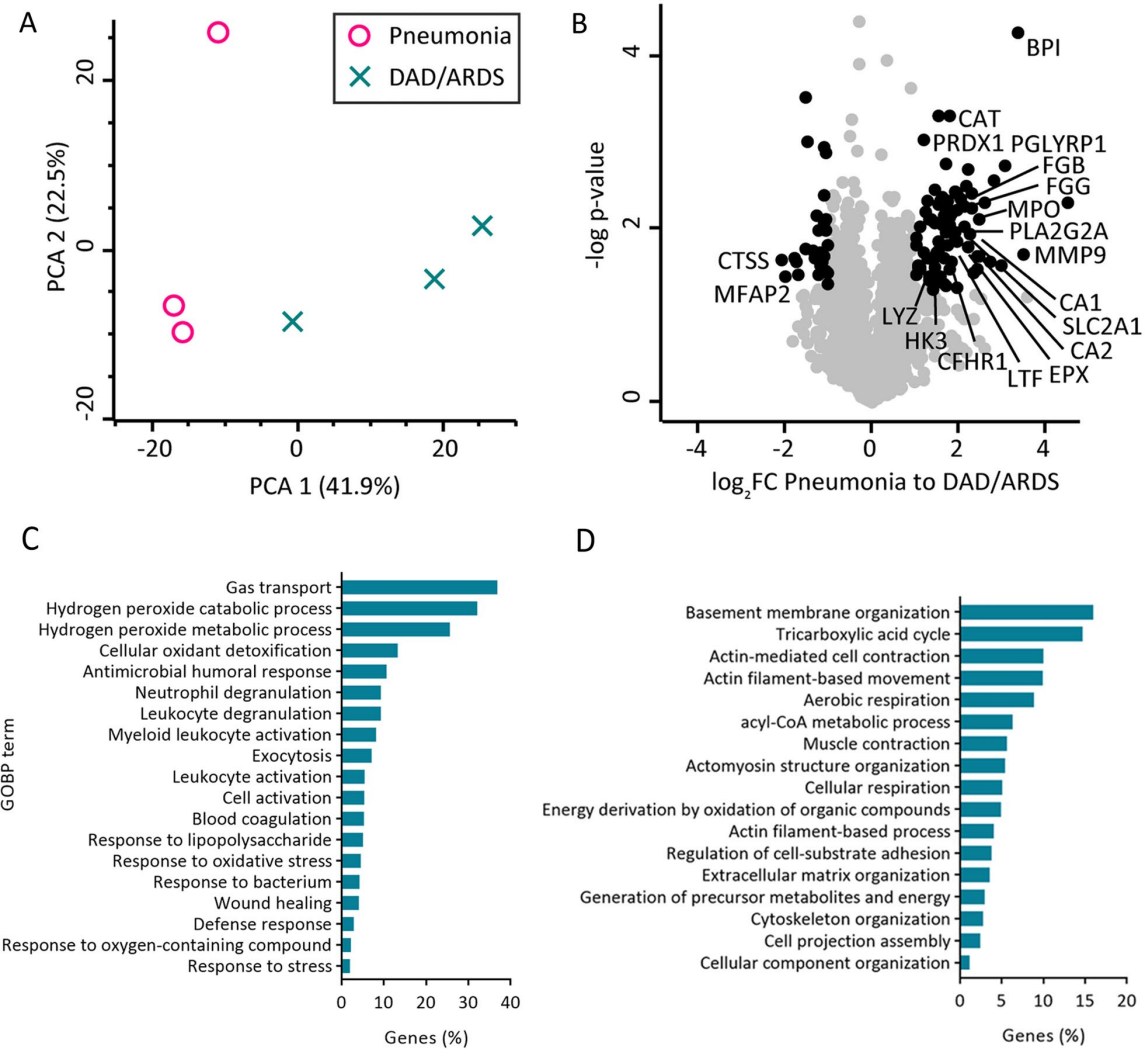
Proteomic profile of lung tissue from COVID-19 patients differs based on the cause of death: pneumonia or DAD/ARDS. **A** Principal component analysis of the COVID-19 samples demonstrates that samples cluster depending on whether the patients died of pneumonia or DAD/ARDS; **B** proteomic profiles correspond to the cause of death, with major bacterial infection response proteins upregulated in pneumonia samples and not in DAD/ARDS. **C** Gene ontology analysis of biological processes (GOBP) of significantly more abundant proteins in the pneumonia cohort (p-value < 0.05, log_2_ fold change (to DAD/ARDS) > 0.5). **D** GOBP of significantly more abundant proteins in the DAD/ARDS cohort (p-value < 0.05, log_2_ fold change (to pneumonia) > 0.5)

Out of the six end-stage COVID-19 patients involved in this study, for three (50%) the pathologically ascribed cause of death was bacterial (secondary) pneumonia (Table 1 and Additional file 2), while for the other three it was diffuse alveolar damage (DAD; in one patient DAD was combined with fungal pneumonia; Table 1 and (Additional file 2: Table S1). Interestingly, despite the low sample number, the proteomic profiles reflect these histopathological differences, as can be seen in the principal component analysis (Fig. 5A) as well as in the volcano plot (Fig. 5B; significantly changed proteins before multi-testing correction marked in black; p-value < 0.05).

Among the proteins upregulated in the pneumonia cohort were mainly proteins involved for leucocyte and neutrophil activation and general response to bacterial infection (e.g. lysozyme C (LYZ), bactericidal permeability-increasing protein (BPI), peptidoglycan recognition protein 1 (PGLYRP1) and others), as well as oxidative stress defence (e.g. catalase (CAT), peroxiredoxin-1 (PRDX1), Myeloperoxidase (MPO); (Additional file 6: Table S5). This was also corroborated by GOBP enrichment analysis of the significantly more abundant proteins in the pneumonia cohort as input (p-value < 0.05, log_2_ fold change (compared to DAD) > 0.5; Fig. 5C). In addition, in the pneumonia cohort also a wound repair response can be observed (Fig. 5C). However, as expected, more prominent ECM and basement membrane reorganization was detected in the DAD group (Fig. 5D). In addition, cytoskeleton organization as well as cellular respiration was prominently affected in DAD samples. A complete list of enriched GOBP terms is included in (Additional file 7: Table S6).

## Discussion

SARS-CoV-2 enters the host through the angiotensin-converting enzyme 2 (ACE2) receptor, which is expressed in various human organs, including lungs [38]. An early COVID-19 proteomics study of formalin-fixed paraffin-embedded lung tissue from the original Wuhan patients reported higher expression of ACE2, cathepsins B and L and a panel of S100 proteins [39] in SARS-CoV-2 affected lungs. Although we could not detect ACE2 in our set of tissue samples, we did also observe a trend towards higher abundance of cathepsins (both B and L) as well as S100 inflammatory mediators in COVID-19 lungs (including S100A8, 9, 11, 12 and P, as reported by [39]) (Additional file 3: Table S2). We also saw upregulation of a number of other inflammatory mediators, including different interferon-induced proteins, inter-alpha-trypsin inhibitor family (ITIH) members, different isoforms of alpha-1-acid glycoprotein and other.

Furthermore, in addition to prominent inflammation in COVID-19 affected lungs, in our dataset we also observed changes in proteins linked to other pathologies, including clot formation, fibrinolysis as well as prominent ECM deregulation (Fig. 1).

It is known that the most common pathological feature in the lungs of COVID-19 diseased patients is DAD, often followed by additional lung injury in form of secondary pneumonia [24, 40, 41]. DAD is known to coincide with excessive ECM remodelling, mainly in the area of the alveolar septa [42]. Prolonged DAD can result in a fibrotic phenotype, typically manifested through loss of the protective epithelial barrier, disruption of laminin-rich basement membrane and secretion of ECM proteins [43, 44]. Correspondingly and in accordance to previously published results [45, 46], we observed a prominent downregulation of proteins vital for proper ECM organisation in diseased lungs. We further corroborate (on both molecular as well as on histopathological level) that ECM is severely deregulated in fatal COVID-19 patients (Figs. 2 and 3). Furthermore, we report that the basement membrane is indeed compromised as a consequence of advanced SARS-CoV-2 infection (as described by [26]), with reduced expression of cadherins responsible for maintaining the integrity of tight junctions (Additional file 1: Fig. S2 and Additional file 3: Table S2, respectively) as well as concomitant loss of laminin and tropomyosin expression (Figs. 2A and 3, Additional file 3: Table S2). The observed increase in clot formation in COVID-19 patients might also be a consequence of these microvascular injuries [47].

However, although lungs are facing an acute injury, tissue repair mechanisms in fatal COVID-19 cases seem to be defective. Signalling pathways responsible for tissue repair, mitosis and cellular proliferation are shut down in COVID-19 lung tissue, while acute response and PTEN signalling are activated (Fig. 4). This is also a conceivable reason for the poor recovery of a significant number of patients who experience lasting negative effects over the timeframe of several month [48]. Deactivation of proliferative signalling was already described for an in vitro time-course experiment of SARS-CoV-2 infection [31]. Similarly, PTEN activation (followed by silencing of other signalling pathways) was also reported upon RNA sequencing of tracheal aspirate from severe COVID-19 ARDS patients [49]. The same authors also report a data-predicted deactivation of different interleukin pathways in COVID-19 patients compared to non-COVID-19 controls, which they attribute to dexamethasone treatment and which was observed in our study too.

Lastly, we demonstrate that COVID-19 related comorbidities seem to be also reflected in the lung proteome. Patients that died due to a secondary pneumonia display an upregulation of proteins involved in response to bacterial infection, immune response and antioxidative defense. The DAD sub-cohort, however, had an increase in abundance of proteins involved in basement membrane and ECM restructuring, as expected in the aftermath of DAD related lung tissue injury. While the subgroup analysis was carried out on a smaller sample number and therefore harbors lower statistical power, it demonstrates that histopathological patient stratification is important when interpreting molecular COVID-19 data.

Collectively, our data represents a comprehensive overview of pathological changes of lung tissue related to terminal COVID-19, on both molecular and histopathological level. Although the study was carried out at a single time point on a relatively small patient cohort with varying time intervals between infection and death, due to the technically challenging collection in a biosafety level three autopsy facility, and as such has its limits, we are still in progress of learning about this disease. Considering all the ongoing challenges to tackle the COVID-19 pandemic long term, all information that can be obtained is invaluable. Data collected in this study provides a comprehensive foundation for further fundamental as well as drug discovery studies on COVID-19.

## Supplementary Information

**Additional file 1: Figure S1.** Overview of the diaPASEF proteomics datasets and **Figure S2. **Cadherin expression is reduced in COVID-19 patients.

**Additional file 2: Table S1.** Patient metadata.

**Additional file 3: Table S2.** output of the limma analysis of the proteomics data.

**Additional file 4: Table S3.** String and Reactome enrichment analysis output.

**Additional file 5: Table S4.** IPA canonical pathways and diseases analysis output.

**Additional file 6: Table S5.** List of differentially abundant proteins in Pneumonia *versus* ARDS.

**Additional file 7: Table S6.** GO enrichment analysis of proteins differentially expressed in Pneumonia.

## Data Availability

The datasets supporting the conclusions of this article (including the DIA-NN version used to process the data) are available at the ProteomeXchange Consortium via the PRIDE partner repository [15] with the dataset identifier PXD030009.

## Abbreviations

ACE2: Angiotensin-converting enzyme 2
AKT1/2: RAC-alpha serine/threonine-protein kinase 1 and 2
ARDS: Acute respiratory distress syndrome
BPI: Bactericidal permeability-increasing protein
CAT: Catalase
COVID-19: Coronavirus disease
DAD: Diffuse alveolar damage
diaPASEF: Parallel accumulation–serial fragmentation combined with data-independent acquisition
ECM: Extracellular matrix
F12: Coagulation factor XII
F2: Prothrombin
FDR: False discovery rate
GC: Vitamin D binding protein
GOBP: Gene ontology enrichment of biological processes
ICU: Intensive care unit
IL: Interleukin
IPA: Ingenuity pathway analysis
ITIH: Inter-alpha-trypsin inhibitor
LYZ: Lysozyme C
MHC: Major histocompatibility complex
MPO: Myeloperoxidase
mTOR: Mammalian target of rapamycin
MX1: Interferon-induced GTP-binding protein Mx1
PGLYRP1: Peptidoglycan recognition protein 1
PI3K: Phosphoinositide-3-kinase
PLG: Plasminogen
PRDX1: Peroxiredoxin-1
PRKACA/B: CAMP-dependent protein kinase A/B
PTEN: Phosphatase and tensin homolog
ROCK1/2: Rho-associated protein kinase 1/2
SERPINC1: Antithrombin-III
STAT: Signal transducers and activators of transcription
TIMS: Trapped ion mobility spectrometry
