# Supplementary material for: Proteomic profiling of end-stage COVID-19 lung biopsies
Source: Clin Proteomics. 2022 Dec 17;19:46. doi: 10.1186/s12014-022-09386-6 (PMC9758034; doi:10.1186/s12014-022-09386-6)
Supplement: Supplementary file 1 — Additional file 1: Figure S1. Overview of the diaPASEF proteomics datasets and Figure S2. Cadherin expression is reduced in COVID-19 patients. [file 12014_2022_9386_MOESM1_ESM.docx]

**Proteomic profiling of end-stage COVID-19 diseased human lungs**

Juergen Gindlhuber^1#^, Tamara Tomin^2#^, Florian Wiesenhofer^3,4^, Martin Zacharias^1^, Laura Liesinger^1^, Vadim Demichev^5^, Klaus Kratochwill^3,4^, Gregor Gorkiewicz^1^, Matthias Schittmayer^2*^ and Ruth Birner-Gruenberger^1,2*^

^#^ authors contributed equally and should be considered co-first authors

^1^ Diagnostic and Research Institute of Pathology, Medical University of Graz, Graz, Austria

^2^ Institute of Chemical Technologies and Analytics, Faculty of Technical Chemistry, Vienna University of Technology-TU Wien, Vienna, Austria

^3^ Christian Doppler Laboratory for Molecular Stress Research in Peritoneal Dialysis, Department of Pediatrics and Adolescent Medicine, Medical University of Vienna, Vienna, Austria

^4^ Division of Pediatric Nephrology and Gastroenterology, Department of Pediatrics and Adolescent Medicine, Comprehensive Center for Pediatrics, Medical University of Vienna, Vienna, Austria

^5^ Institute of Biochemistry, Charité - Universitätsmedizin Berlin, Berlin, Germany

* Correspondence: Matthias Schittmayer ([matthias.schittmayer@tuwien.ac.at](mailto:matthias.schittmayer@tuwien.ac.at)) and Ruth Birner-Gruenberger ([ruth.birner-gruenberger@tuwien.ac.at](mailto:ruth.birner-gruenberger@tuwien.ac.at))

# Supplementary Figures and Tables


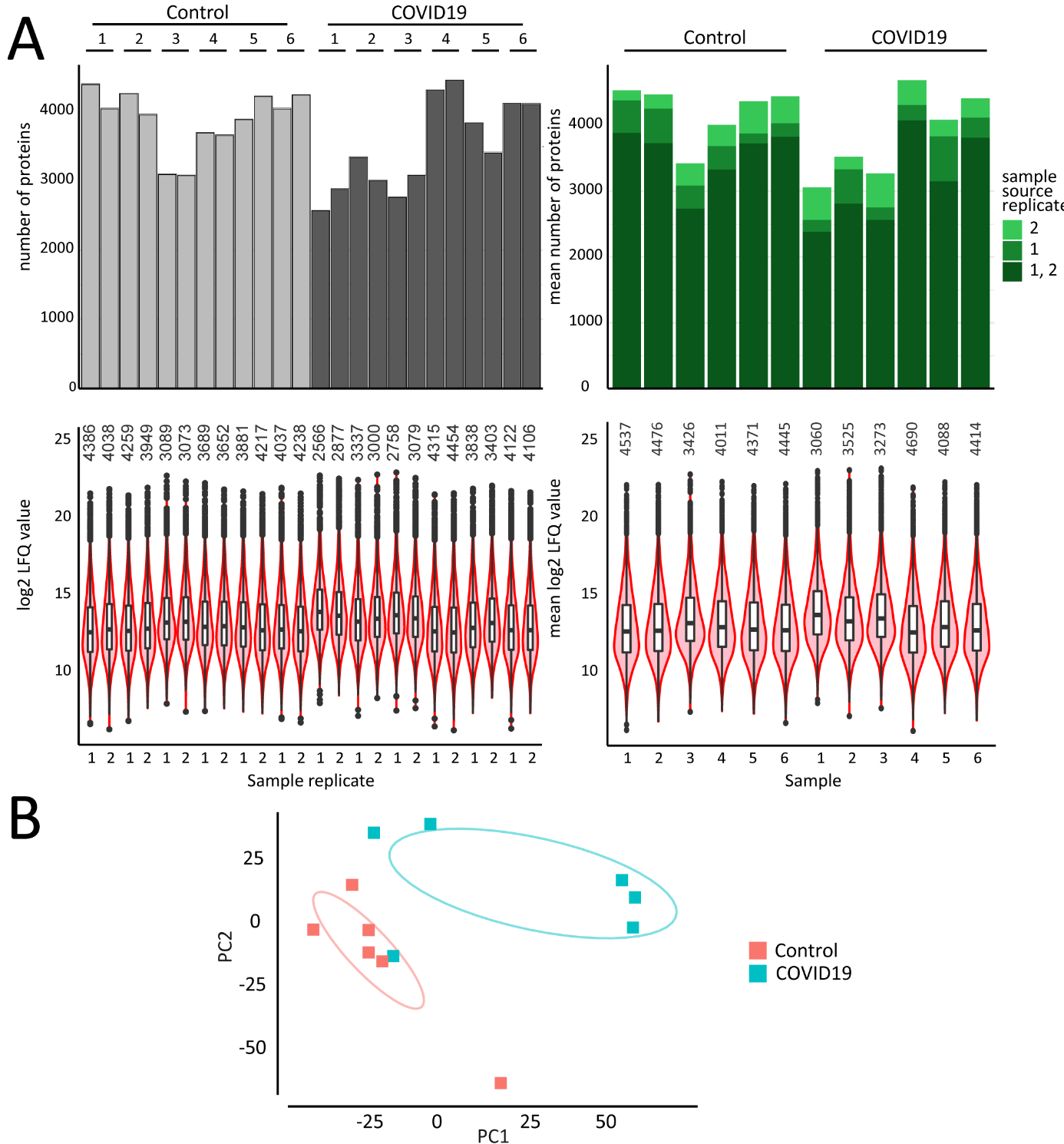


**Figure S1. Overview of the diaPASEF proteomics datasets**. A: From each patient (control or COVID-19) two individual lung tissue samples (replicates) were taken at the point of autopsy (left). The two replicates were processed and measured separately and the resulting data was merged per sample by taking a mean value from the two replicates per each protein, when possible (right); B: Principal component analysis of the COVID19 versus the control group.

***
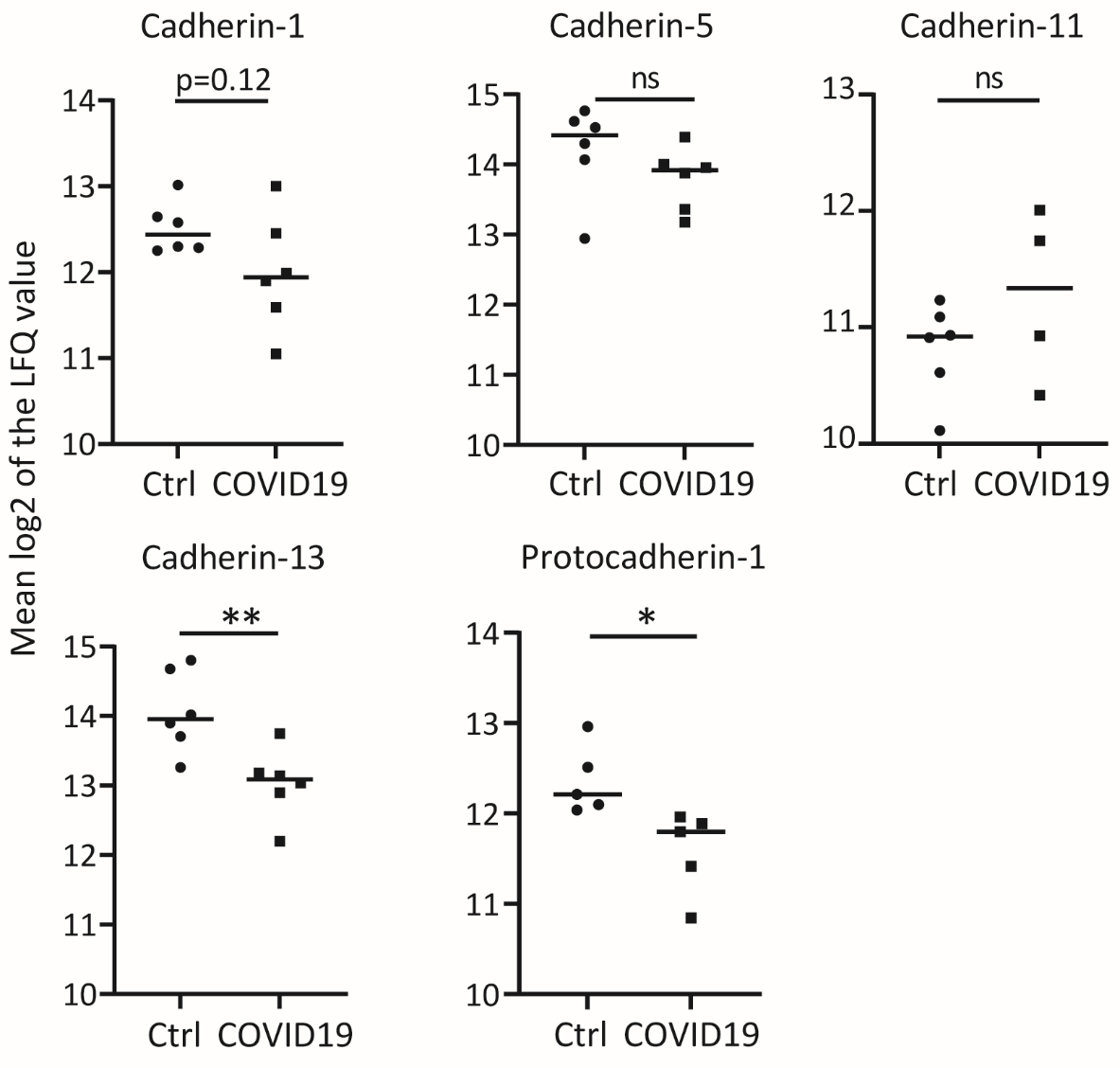
***

**Figure S2. Cadherin expression is reduced in COVID-19 patients**. N=6 per group, * Student’s t-test p-value < 0.05; ** Student’s t-test p-value < 0.01; ns – not significant.
